# Amine-containing yolk-shell structured magnetic organosilica nanocomposite as a highly efficient catalyst for the Knoevenagel reaction

**DOI:** 10.3389/fchem.2024.1336855

**Published:** 2024-02-06

**Authors:** Maryam Neysi, Dawood Elhamifar

**Affiliations:** Department of Chemistry, Yasouj University, Yasouj, Iran

**Keywords:** nanocatalyst, magnetic nanoparticles, mesoporous organosilica, yolk-shell structured nanocomposite, Knoevenagel reaction

## Abstract

The yolk-shell structured silica nanocomposites have been considered by many researchers due to their specific physical and chemical properties. These materials have been widely used in adsorption and catalysis processes. Especially, the void space of yolk−shell nanostructures can provide a unique environment for storage, compartmentation, and confinement in host−guest interactions. In this paper, for the first time, the preparation, characterization, and catalytic application of a novel amine-containing magnetic methylene-based periodic mesoporous organosilica with yolk-shell structure (YS-MPMO/pr-NH_2_) are developed. The magnetic periodic mesoporous organosilica nanocomposite was synthesized through surfactant-directed co-condensation of bis(triethoxysilyl)methane (BTEM) and tetraethoxysilane around Fe_3_O_4_ nanoparticles. After Soxhlet extraction, the surface of YS-MPMO nanocomposite was modified with 3-aminopropyl trimethoxysilane to deliver YS-MPMO-pr-NH_2_ nanocatalyst. This catalyst was characterized by using EDX, FT-IR, VSM, TGA, XRD, nitrogen-sorption, and SEM analyses. The catalytic activity of YS-MPMO/pr-NH_2_ was studied in the Knoevenagel reaction giving the corresponding products in a high yield and selectivity. The YS-MPMO/pr-NH_2_ nanocatalyst was recovered and reused at least four times without a significant decrease in efficiency and activity. A leaching test was performed to study the nature of the catalyst during reaction conditions Also, the catalytic performance of our designed nanocomposite was compared with some of the previous catalysts used in the Knoevenagel reaction.

## 1 Introduction

In recent years, silica-based nanocomposites have received much attention between researchers in various fields of chemistry. These materials have been extensively employed in chemical processes due to the good properties of silica such as high chemical and thermal stability, high colloidal stability, biocompatibility and easy surface modification (Maleki et al., 2015; Purbia and Paria, 2015; Sun et al., 2015; Cheng et al., 2017; Wang et al., 2019; Gopalan Sibi et al., 2020). Among these, yolk-shell (YS) structured silica nanocomposites have been considered and studied by many researchers (Nagaraju et al., 2017; Bai et al., 2018; Du et al., 2018). These nanocomposites have many applications in the areas of drug delivery, catalysis, charge transfer and storage in batteries, solar cells and supercapacitors, adsorbents for gases and pollutants, gene therapy, etc (Nagaraju et al., 2017; Xie et al., 2017; Bai et al., 2018; Du et al., 2018). For example, recently, the YS-structured nanocomposites have been used as catalyst in the synthesis of pyranopyrazoles (Neysi and Elhamifar, 2023), the Chan-Lum coupling reaction (Shaker and Elhamifar, 2021), and the reduction of nitrobenzenes (Wang et al., 2018).

Among the various types of yolk-shell structured silica nanocomposite, those that are composed of Fe_3_O_4_ core and PMO shell have been highly regarded by researchers due to their unique magnetic response, high adsorption capacity, high surface area, and high hydrophobicity (Haffer et al., 2010; Croissant et al., 2014; Lu et al., 2016; Wei et al., 2016; Abaeezadeh et al., 2019; Yu L. et al., 2019; Liu et al., 2019; Kargar et al., 2020). These nanocomposites have been used in various fields such as biomedicine, battery development, fuel cell technology, sensor technology, gene therapy, and nanocatalysis (Li H. et al., 2018; Li J. et al., 2018; Lin et al., 2018; Wang et al., 2018; Li X.-P. et al., 2019; Yu K. et al., 2019; Zhang et al., 2019). Some of recently reported nanocomposites with Fe_3_O_4_ core and PMO shells are Fe_3_O_4_@SiO_2_@PMO (Mirbagheri et al., 2021), Fe_3_O_4_-YS-PMO (Wei et al., 2016), YS-Fe_3_O_4_@Au@PMO (Liu et al., 2019), Fe_3_O_4_@mSiO_2_ (Li Y. et al., 2019), Fe_3_O_4_@PMO-NH_2_ (Rosso et al., 2020) and Fe_3_O_4_@MePMO-IL/Pd (Shaker and Elhamifar, 2020).

On the other hand, the Knoevenagel reaction (Gordel-Wojcik et al., 2022) is one of the most famous carbon-carbon coupling process to synthesize α,β-unsaturated compounds. In recent years, the synthesis of the Knoevenagel products in the presence of heterogeneous and homogeneous catalysts has been investigated under different conditions. Due to difficulty in the separation of homogeneous catalysts, the use of magnetic heterogeneous catalysts is a good option to improve the efficiency of the catalytic processes. Some of recently reported studies in this matter are Fe_3_O_4_@SiO_2_@propyl@DBU (Zhang et al., 2021), L-proline-Cu/TCT@NH2@Fe3O4 (Kalantari et al., 2022), MgFe_2_O_4_(Ghomi and Akbarzadeh, 2018) and Fe_3_O_4_–cysteamine hydrochloride (Maleki et al., 2017).

In view of the above, in this research, a novel magnetic yolk-shell structured PMO supported propylamine (YS-MPMO/pr-NH_2_) is prepared, characterized and its catalytic application is developed in the Knoevenagel reaction under green conditions.

## 2 Experimental section

### 2.1 Synthesis of Fe_3_O_4_ nanoparticles

Fe_3_O_4_ NPs were firstly prepared according to our previous procedure (Neysi et al., 2020). According to this method, FeCl_2_.4H_2_O (1.5 g) and FeCl_3_.6H_2_O (3 g) were dissolved in 160 mL of deionized water. Then, aqueous ammonia (40 mL, 28% wt) was slowly added and the obtained mixture was stirred at room temperature (RT) for 60 min under argon atmosphere. The resulting product was collected using an external magnet and it was washed completely with distilled water and EtOH. This product was dried at 70°C for 12 h under vacuum and called Fe_3_O_4_ nanoparticles.

### 2.2 Preparation of yolk-shell structured magnetic PMO (YS-MPMO)

To prepare of YS-MPMO, firstly, Fe_3_O_4_ NPs (1 g) were completely dispersed in H_2_O (20 mL). Then, this mixture was added to a reaction vessel containing H_2_O (36 mL), EtOH (16 mL), cetyltrimethylammonium bromide (CTAB, 0.72 g), pluronic P123 (1.7 g) and ammonia (0.9 mL, 25% wt). The obtained combination was stirred at 35°C–40°C for 30 min. Next, 1,2-bis(triethoxysilyl)methane (BTEM, 2.1 g) and tetraethoxysilane (TEOS, 0.7 g) were added while stirring under the same conditions for 1 h. After that, the resulting mixture was heated at 100°C for 17 h under static conditions. Finally, the product was magnetically separated, washed with EtOH and H_2_O and dried. The surfactants were removed using a Soxhlet apparatus to give the YS-MPMO product.

### 2.3 Synthesis of YS-MPMO/pr-NH_2_

For this, firstly, the YS-MPMO nanocomposite (1 g) was dispersed in toluene (25 mL) at RT. Then, APTMS (3-aminopropyltrimethoxysilane, 98%, 1 mmol) was added and the resulting mixture was stirred at 100°C for 24 h. In the following, the product was magnetically separated, washed with EtOH and H_2_O, dried at 60°C for 12 h and called YS-MPMO/pr-NH_2_ nanocomposite. According to the CHN and EDX analyses the loading of amine groups on the designed nanocomposite surface was found to be 0.5 mmol/g.

### 2.4 Procedure for Knoevenagel reaction

For this, aldehyde (1 mmol), malononitrile (1 mmol) and YS-MPMO/pr-NH_2_ catalyst (2.25 mol%) were added in a reaction vessel while sonicating under solvent-free conditions at RT. In the end of reaction, monitored by TLC, EtOH (5 mL) was added and catalyst was magnetically removed. Then, the EtOH solvent was evaporated and impure products were recrystallized in EtOH and *n*-hexane solvents to give pure Knoevenagel products.

### 2.5 IR, ^1^H and ^13^C-NMR data of Knoevenagel products

#### 2.5.1 2-(2-Chlorobenzylidene)malononitrile

IR (KBr, cm^−1^): 3035(=C–H, stretching vibration, sp^2^), 2223 (C≡N), 1480–1612 (C=C, Ar stretching sp^2^). ^1^H-NMR (400 MHz, CDCl_3_): δ (ppm), 7.58–7.63 (m, 1H), 7.66–7.75 (m, 1H), 8.06 (d, 1H, J = 6.0 Hz), 8.58 (d, 1H, J = 4.0), 8.70 (s, 1H). ^13^C-NMR (100 MHz, CDCl_3_): δ (ppm), 63.1, 87.14, 112.8, 113.9, 130.3, 130.8, 134.7, 135.3, 159.5.

#### 2.5.2 2-(4-Nitrobenzylidene)malononitrile

IR (KBr, cm^−1^): 3105 (C-H, stretching vibration, sp^2^), 2204 (C≡N), 1514, 1358 (NO_2_, stretching vibration), 1411–1609 (C=C, Ar stretching sp^2^).^1^H-NMR (400 MHz, CDCl_3_): δ (ppm), 7.04 (d, 2H, J = 8.4 Hz), 6.6 (d, 2H, J = 8.4 Hz), 5.23 (s, 1H). ^13^C-NMR (100 MHz, CDCl_3_): δ (ppm), 85.39, 113.08, 114.13, 125.32, 128.40, 136.33, 148.49, 159.74.

## 3 Result and discussion

**FIGURE 1:**
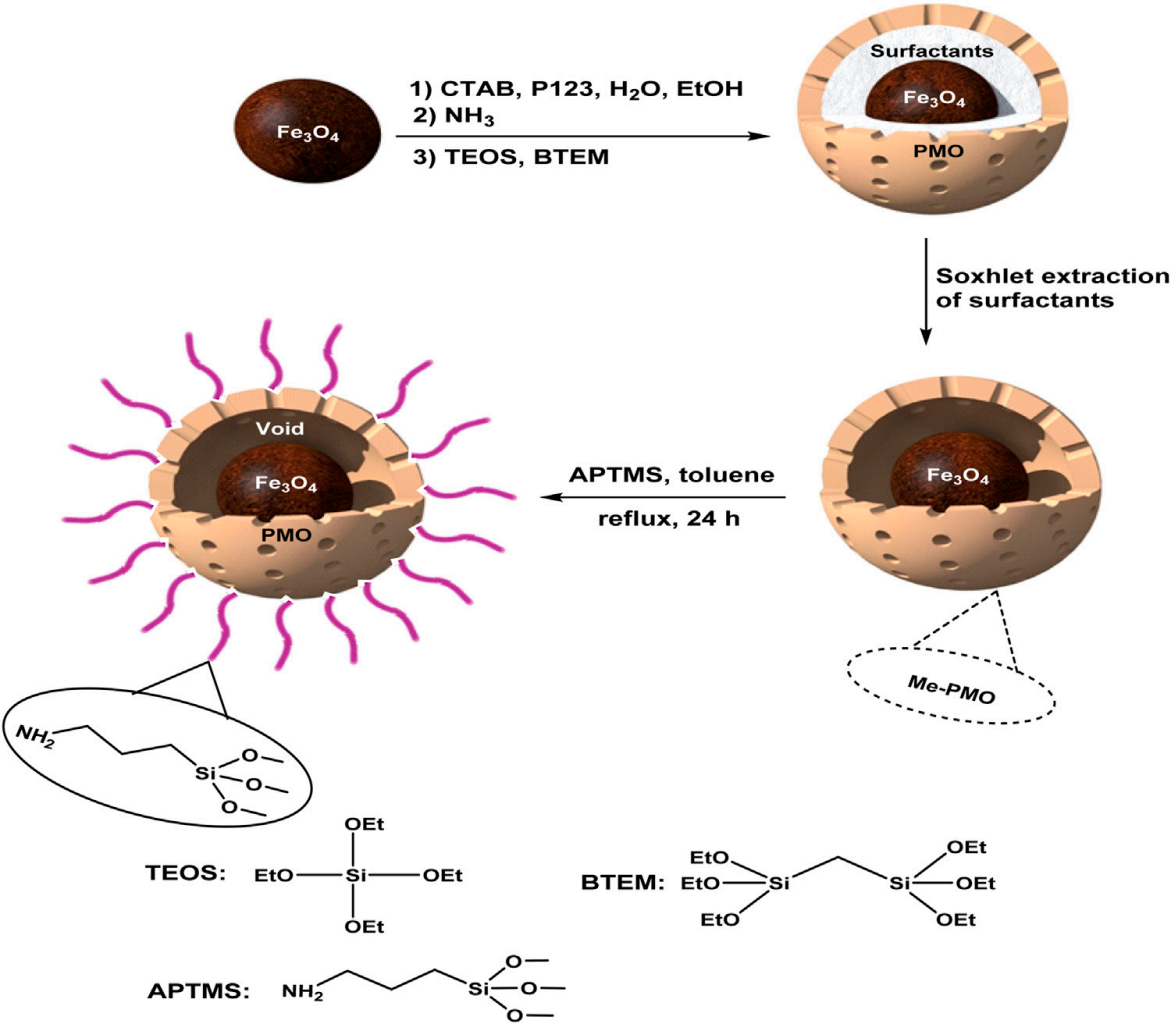
Preparation of the YS-MPMO/pr-NH_2_ nanocomposite.

Firstly, core-shell structured magnetic periodic mesoporous organosilica (MPMO) was synthesized via hydrolysis and co-condensation of BTEM and TEOS around Fe_3_O_4_ NPs in the presence of CTAB and pluronic P123 surfactants. After Soxhlet extraction of surfactants, the YS-MPMO was produced. This material was then modified with 3-aminopropyltrimethoxysilane (APTMS) to give YS-MPMO/pr-NH_2_ nanocomposite (Figure 1).

**FIGURE 2:**
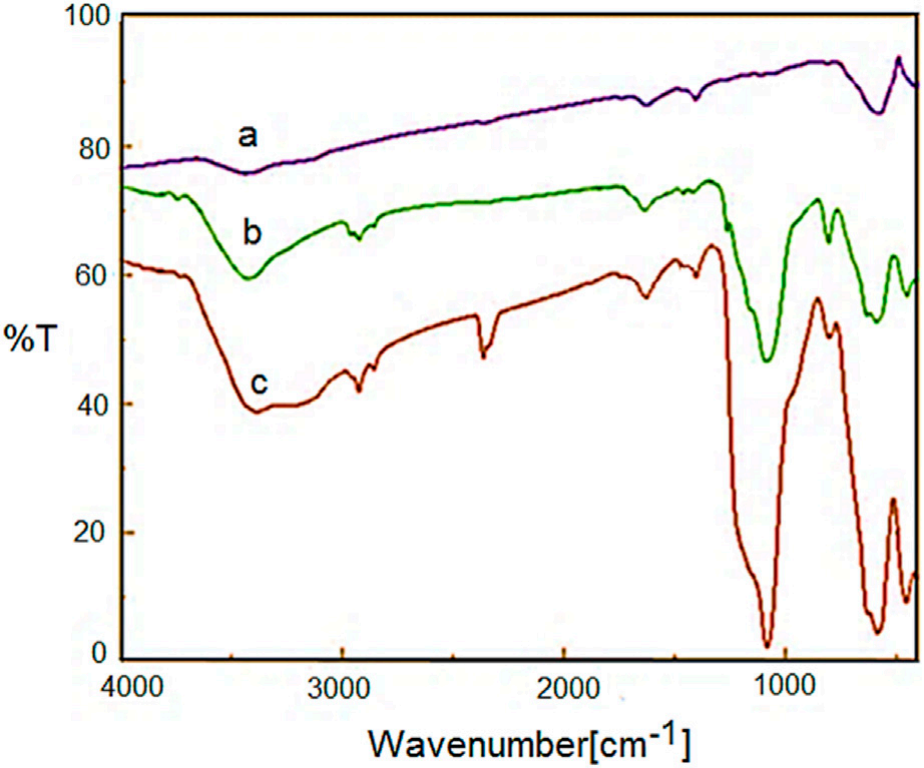
FT-IR spectra of a) Fe_3_O_4_, b) YS-MPMO and c) YS-MPMO/pr-NH_2_.

Figure 2 shows the FT-IR spectra of Fe_3_O_4_, YS-MPMO and YS-MPMO/pr-NH_2_ nanoparticles. For all materials, the characteristic peaks of Fe−O and O-H bonds are, respectively, appeared at 588 and 3,400 cm^−1^ (Figures 2A–C). In the FT-IR spectra of YS-MPMO and YS-MPMO/pr-NH_2_, the peaks at 940 and 1,090 cm^−1^ are, respectively, assigned to symmetric and asymmetric vibrations of the Si-O-Si bonds proving the successful formation of silica layer around the Fe_3_O_4_ NPs. Also, for YS-MPMO and YS-MPMO/pr-NH_2_ nanocomposits, the C-H signals of aliphatic moieties are appeared at 2,880–2,911 cm^−1^ (Figures 2B,C).

**FIGURE 3:**
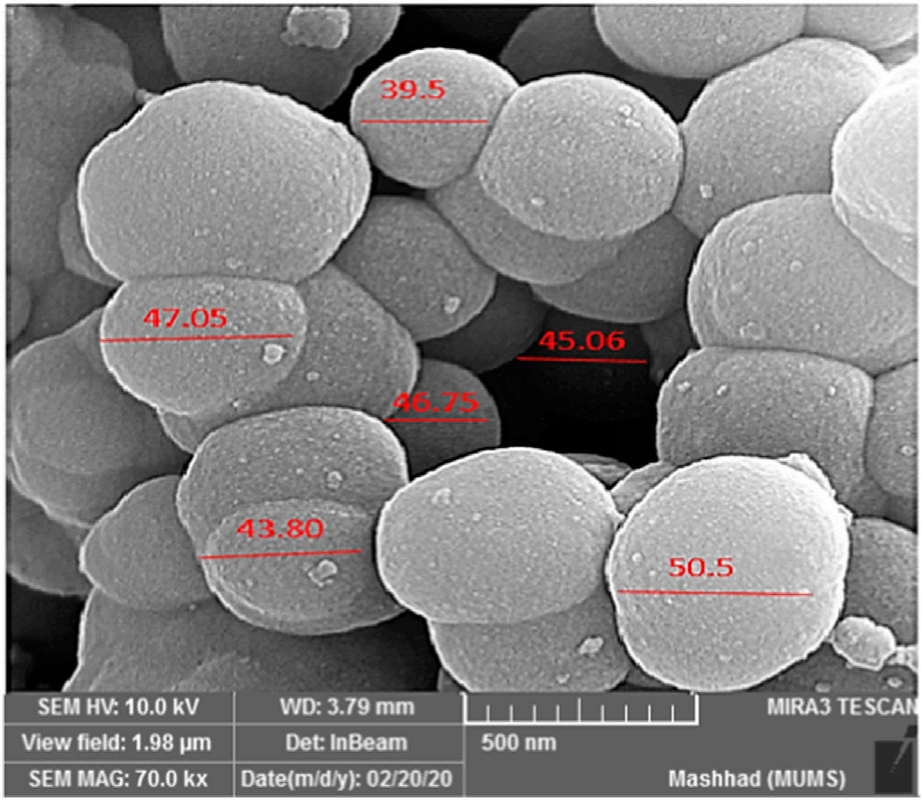
SEM image of the YS-MPMO/pr-NH_2_ nanocomposite.

The SEM analysis of YS-MPMO/pr-NH_2_ demonstrated a morphology with spherical particles and an average size of about 45 nm (Figure 3). These type nanoparticles are very important in the fields of catalysis and adsorption processes.

**FIGURE 4:**
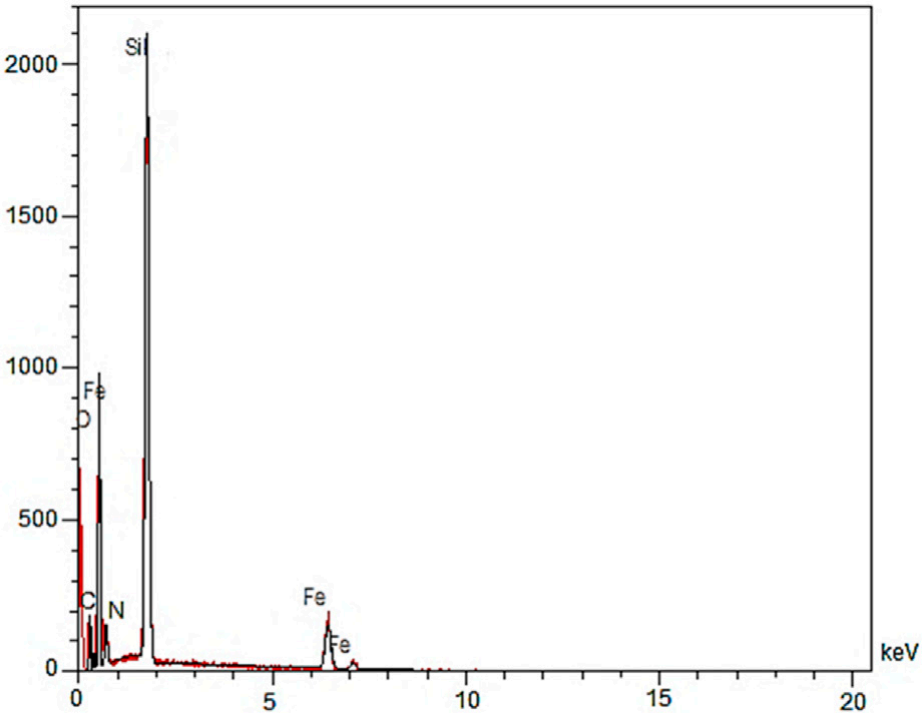
EDX analysis of YS-MPMO/pr-NH_2_.

The EDX analysis of YS-MPMO/pr-NH_2_ nanocomposite successfully confirmed the presence of Fe, O, C, N and Si elements in its framework (Figure 4).

**FIGURE 5:**
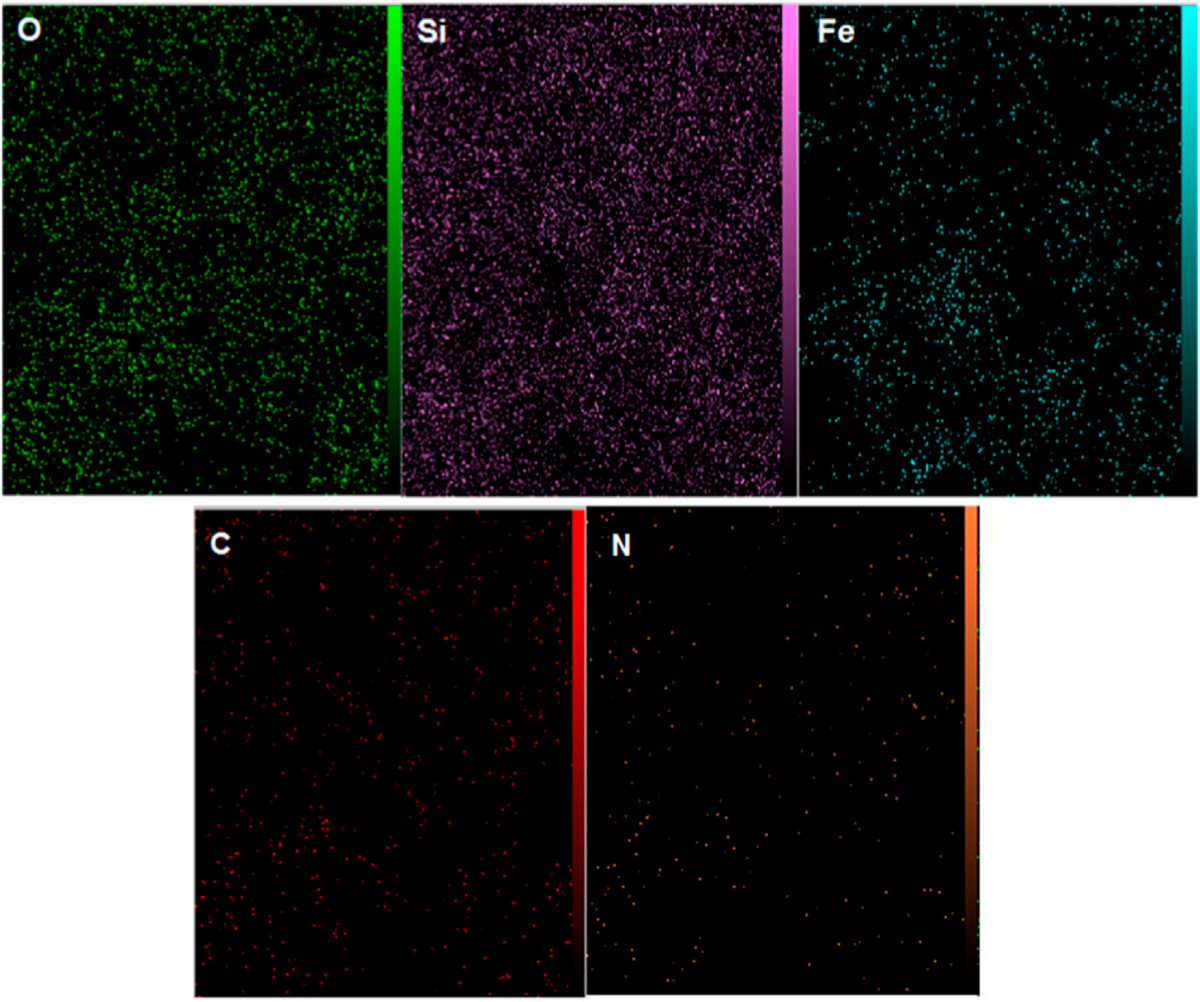
EDX mapping analysis of YS-MPMO/pr-NH_2_.

Also, the EDX mapping analysis revealed the well distribution of aforementioned elements in the framework of the YS-MPMO/pr-NH_2_ nanocomposite (Figure 5). These are in good agreement with the FT-IR results confirming well immobilization/incorporation of methylene and propylamine moieties on/in the material framework.

**FIGURE 6:**
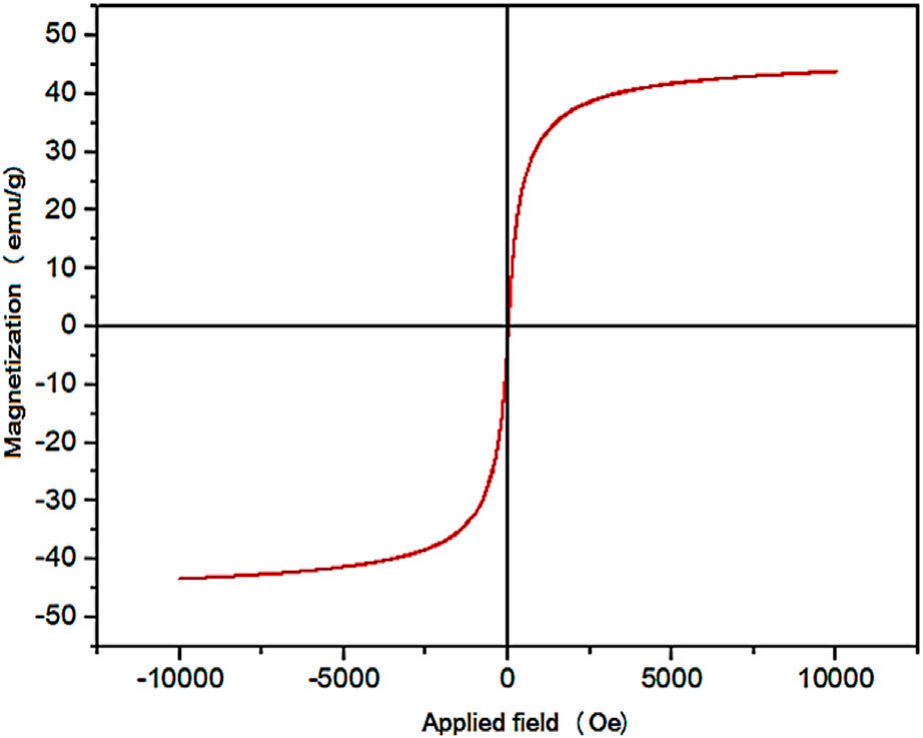
VSM analysis of the YS-MPMO/pr-NH_2_ nanocomposite.

The magnetic properties of YS-MPMO/pr-NH_2_ nanocomposite were evaluated by using VSM analysis. The result of this study showed that YS-MPMO/pr-NH_2_ nanocomposite has a superparamagnetic behavior. Also, the amount of magnetic saturation of this nanocomposite was about 43 emu/g (Figure 6).

**FIGURE 7:**
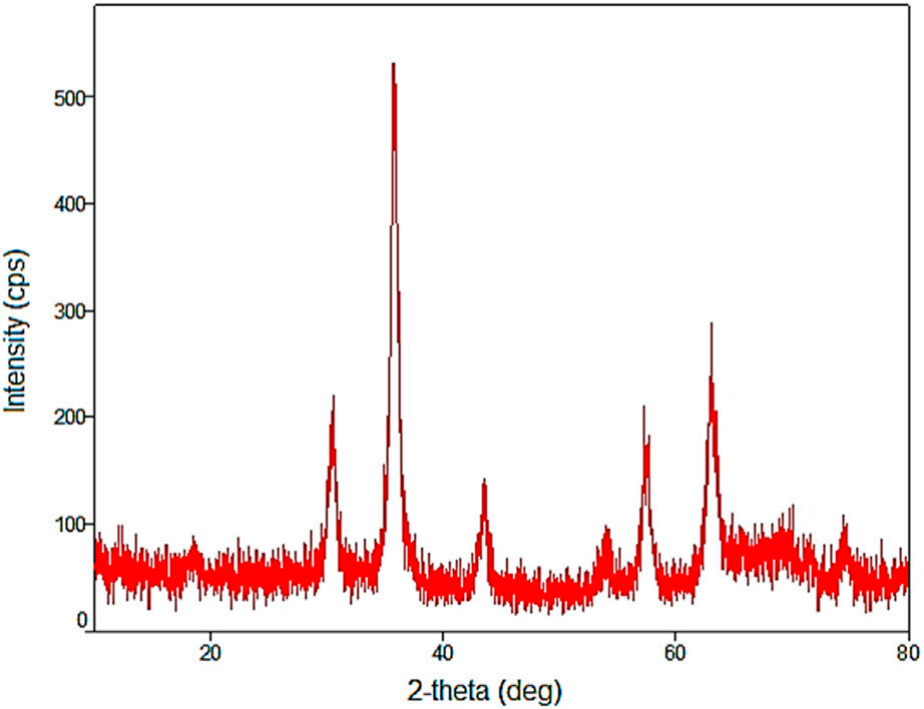
Wide angle PXRD pattern of YS-MPMO/pr-NH_2_.

In the wide angle PXRD pattern of YS-MPMO/pr-NH_2_, the presence of 6 peaks at 2θ: 30.3, 36, 43.5, 54.5, 57.5 and 63°, corresponding to the crystalline structure of Fe_3_O_4_ NPs, affirms the high stability of these nanoparticles during the preparation of the YS-MPMO/pr-NH_2_ nanocomposite (Figure 7).

**FIGURE 8:**
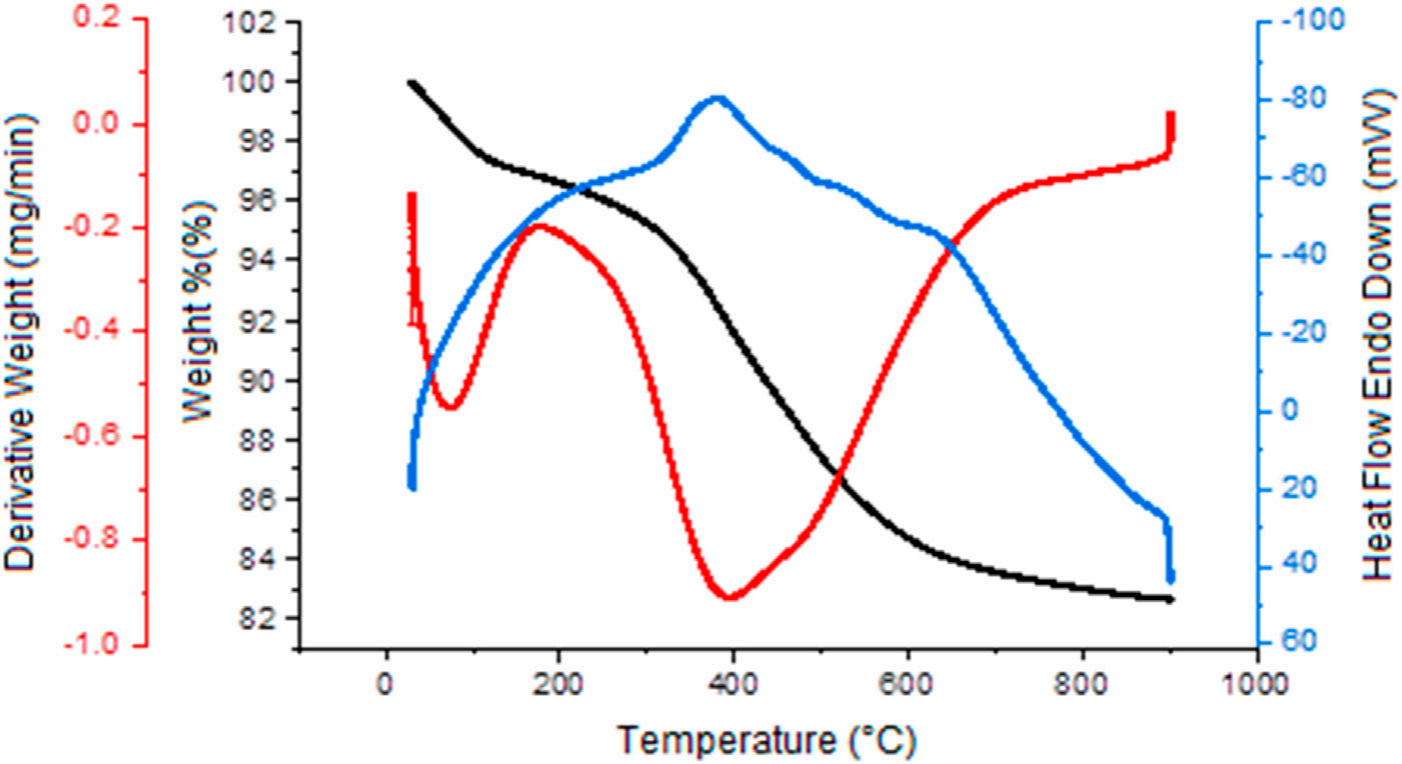
TG analysis of the YS-MPMO/pr-NH_2_ nanocomposite.

The TGA curve of YS-MPMO/pr-NH_2_ nanocomposite showed three weight losses. The first one (about 2%) in the range of 25°C–130°C is assigned to removal of water and organic solvents. The second one (about 2%) at 150°C–280°C is due to the elimination of remained CTAB and pluronic P123 surfactants. The third one at 300°C–700°C (about 11%) is corresponded to the removal of grafted propylamine moieties on the shell surface and also incorporated methylene groups in the shell framework (Figure 8) (Neysi and Elhamifar, 2023).

**FIGURE 9:**
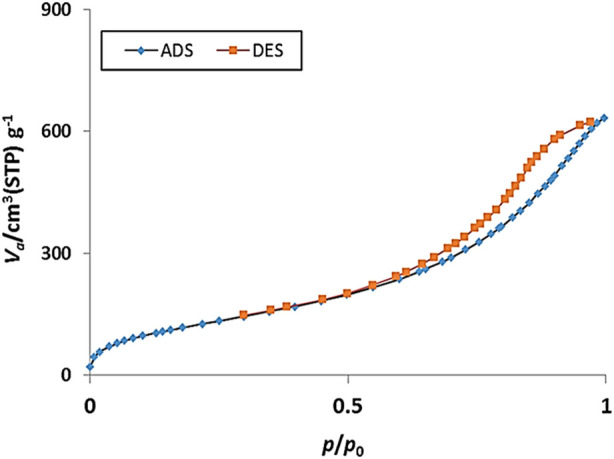
Nitrogen adsorption-desorption isotherm of YS-MPMO/pr-NH_2_.

The N_2_ adsorption-desorption analysis of the YS-MPMO/pr-NH_2_ showed a type IV isotherm with a H_2_ hysteresis loop, corresponding to ordered mesostructured PMO shell (Figure 9). According to this analysis, the BET surface area and pore volume of the nanocomposite were found to be 470.67 m^2^/g and 0.973 cm^3^/g, respectively.

After characterization of the YS-MPMO/pr-NH_2_ nanocomposite, its catalytic activity was examined in the Knoevenagel condensation under ultrasonic conditions. To optimize the reaction conditions, the condensation between malononitrile and benzaldehyde was selected as a model reaction. Examination of the amount of catalyst in this reaction showed that the best yield is obtained in the presence of 2.25 mol% of YS-MPMO/pr-NH_2_ (Table 1, entries 1–4). Next, the catalytic activity of YS-MPMO/pr-NH_2_ was investigated in different solvents of H_2_O, EtOH and toluene and also solvent-free media. This study showed that the best result is obtained under solvent-free conditions. (Table 1, entry 3 vs. entries 5–7). The H-bonding between protic EtOH and water solvents and malononitrile is a parameter which prevents and restricts the activity of this nucleophile in these solvents. Finally, the catalytic activity of amine-free Fe_3_O_4_ and YS-MPMO materials were studied, in which only a little yield of the desired product was obtained confirming that the designed Knoevenagel reaction is catalyzed by supported propylamine groups (Table 1, entry 3 vs. entries 8, 9). Accordingly, the use of 2.25 mol% of catalyst, RT and solvent-free media were selected as optimal conditions (Table 1, entry 3).

**TABLE 1:** The effect of solvent and catalyst loading in the Knoevenagel reaction of malononitrile with benzaldehyde^a^.

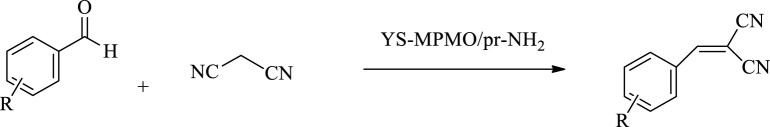				
Entry	Catalyst	Cat. (mol%)	Solvent	Yield (%)
1	YS-MPMO/pr-NH_2_	0.75	-	48
2	YS-MPMO/pr-NH_2_	1.5	-	73
3	YS-MPMO/pr-NH_2_	2.25	-	97
4	YS-MPMO/pr-NH_2_	3	-	97
5	YS-MPMO/pr-NH_2_	2.25	Toluene	60
6	YS-MPMO/pr-NH_2_	2.25	EtOH	83
7	YS-MPMO/pr-NH_2_	2.25	H_2_O	86
8	Fe_3_O_4_	0.015 g	-	<10
9	YS-MPMO	0.015 g	-	<10

^a^ Reaction conditions: Benzaldehyde (1 mmol), ethylcyanoacetate (1 mmol), RT, 70 min.

In the following, the catalytic activity of YS-MPMO/pr-NH_2_ nanocatalyst was investigated in the condensation of various aldehydes with malononitrile under the optimal conditions. The study demonstrated that all aldehydes, bearing both electron withdrawing and electron donating substituents in various positions, give the corresponding Knoevenagel products in high yield and selectivity (Table 2). This confirms the high efficiency of the designed catalyst for the preparation of a wide range of important Knoevenagel products.

**TABLE 2:** Synthesis of the Knoevenagel products in the presence of the YS-MPMO/pr-NH_2_ nanocatalyst.

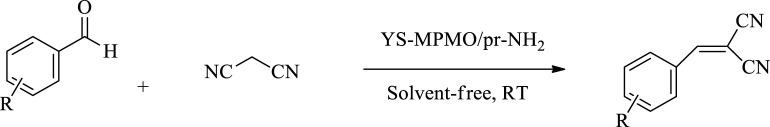							
Entry	Aldehyde	Time (min)	Yield^a^ (%)	TON^b^	TOF^c^	M. P.	Ref.
1	PhCHO	70	97	4311	3717	50–53	50–51 (Heravi et al., 2006)
2	4-NO_2_-PhCHO	65	94	4178	3869	170–173	170–171 (Karimkhah et al., 2021)
3	4-Me-PhCHO	85	92	4089	2900	93–95	93–94 (Heravi et al., 2006)
4	4-Cl-PhCHO	80	93	4133	3107	88–90	87–89 (Heravi et al., 2006)
5	4-OH-PhCHO	90	87	3867	2578	171–173	170–171 (Karimkhah et al., 2021)
6	2-NO_2_-PhCHO	70	93	4133	3563	99–100	98–100 (Karimkhah et al., 2021)
7	4-Br-PhCHO	75	95	4222	3378	89–91	90–91 (Zhang et al., 2017)
8	2-Cl-PhCHO	90	89	3956	2637	52–54	52–54 (Kolahdoozan et al., 2013)

^a^ Isolated yield.

^b^ Turnover number [defined as yield (%)/cat. (mmol)].

^c^ Turnover frequency [defined as TON/reaction time (h)].

**FIGURE 10:**
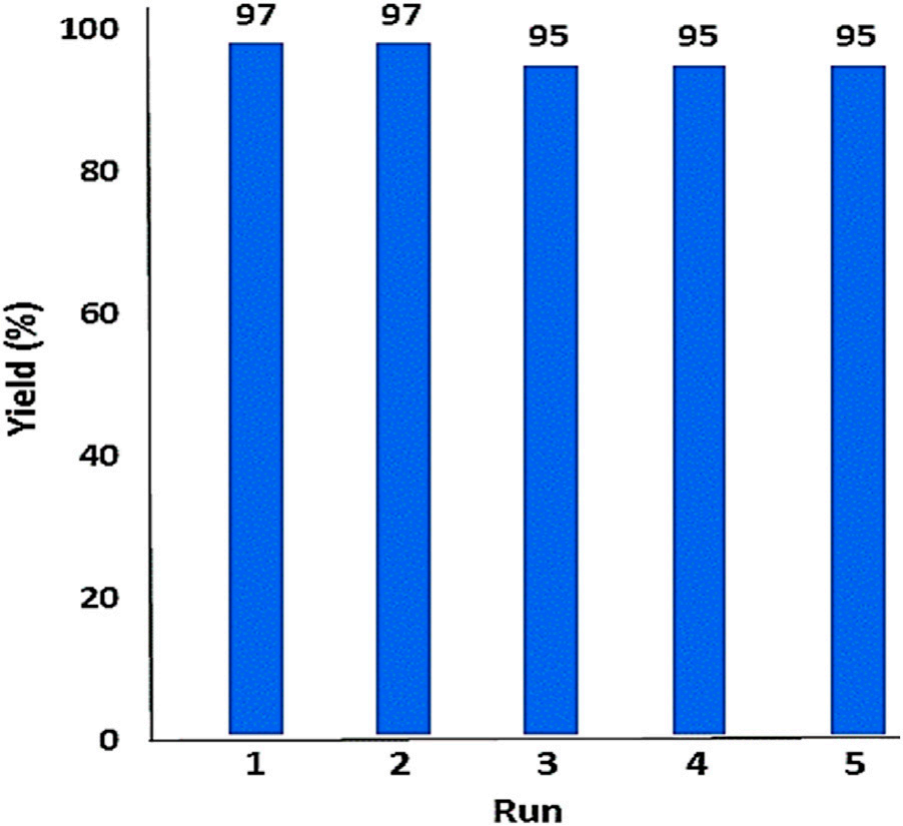
Recoverability and reusability of the YS-MPMO/pr-NH_2_ catalyst.

In the following, the recoverability and reusability of the YS-MPMO/pr-NH_2_ nanocatalyst were investigated in the condensation of malononitrile with benzaldehyde under optimal condition. After completion of the reaction, the catalyst was magnetically removed and reused in the next run under the same conditions as the first run. Based on this study, it was found that YS-MPMO/pr-NH_2_ can be recycled and reused for four runs without a significant decrease in its performance (Figure 10).

Next, a leaching test was performed to study the nature of catalyst under applied conditions. For this, the YS-MPMO/pr-NH_2_ nanocatalyst was added to a flask containing benzaldehyde and malononitrile at RT. After the reaction progressed about 50%, the catalyst was separated using an external magnet and the reaction of residue was monitored for 60 min under optimal conditions. The result demonstrated no further progress of the reaction, confirming no leaching and also heterogeneous nature of active catalytic species under applied conditions.

Finally, a comparison study was performed between the present catalyst and a number of former catalysts applied in the Knoevenagel reaction (Table 3). This showed that YS-MPMO/pr-NH_2_ is better than others in parameters of recovery times, reaction temperature and stability.

**TABLE 3:** Comparison of the catalytic activity of YS-MPMO/pr-NH_2_ with former catalysts.

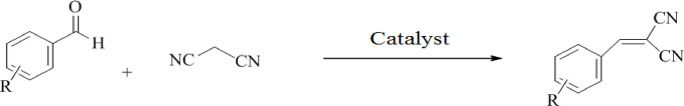					
Entry	Catalyst	Conditions	Time (min)	Recovery times	Ref.
1	RhPt/TC@GO NPs	H_2_O/Methanol, RT	40	2	Şen et al. (2018)
2	Fe_3_O_4_@PMO-ICS–ZnO	EtOH, reflux	60	3	Safapoor et al. (2021)
3	Y_2_ZnO_4_	Solvent-free, under MW (420 W)	15	3	Ghosh et al. (2020)
4	YS-Fe_3_O_4_@PMO/Pr-NH_2_	Solvent free, RT	70	4	This study

**FIGURE 11:**
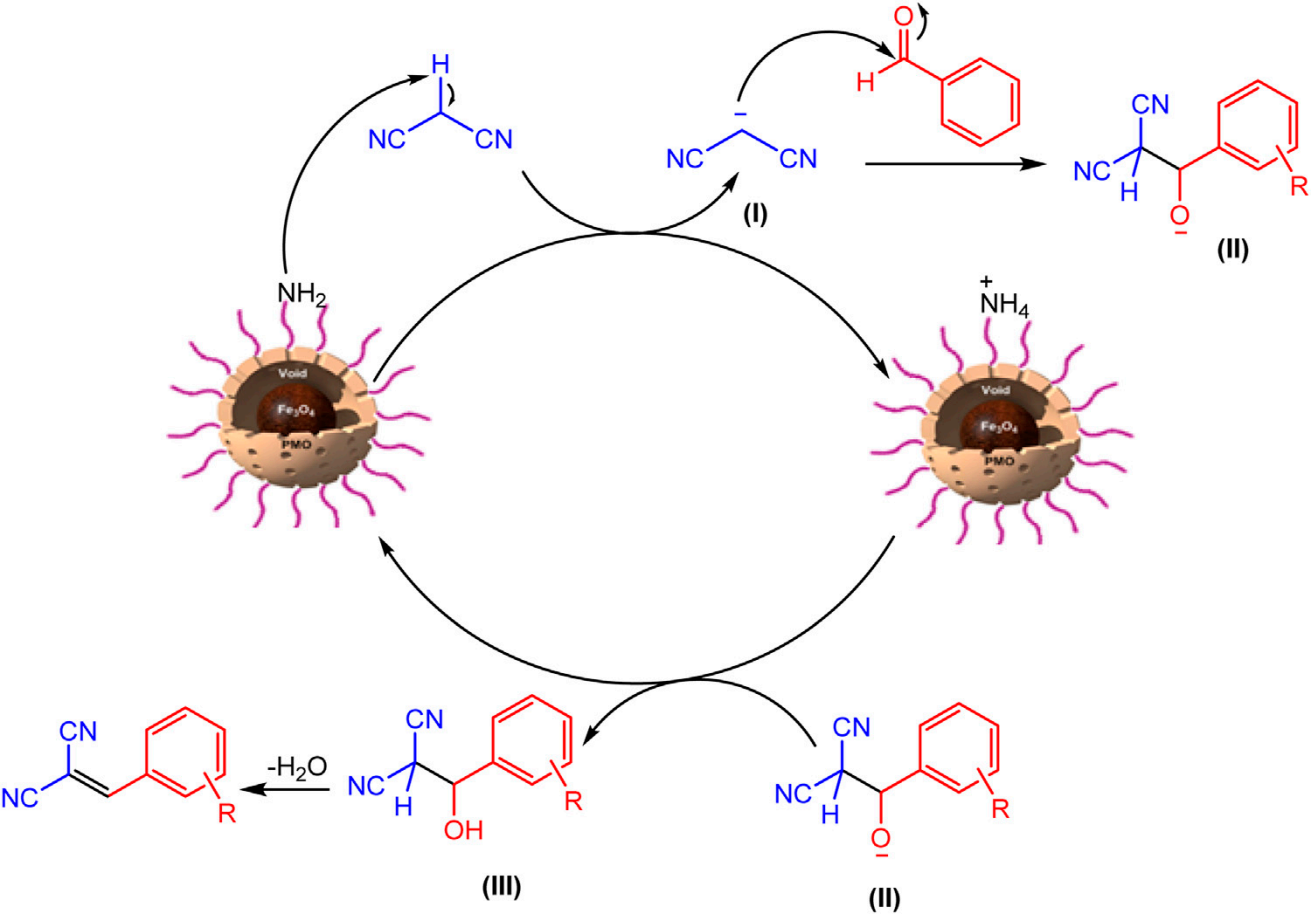
Proposed mechanism for the Knoevenagel condensation using YS-MPMO/pr-NH_2_.

The mechanism of the Knoevenagel reaction is shown in Figure 11. As seen, firstly, one of the active hydrogens of malononitrile methylene is taken by YS-MPMO/pr-NH_2_ nanocatalyst to deliver anion **I**. Then, this anion, as a nucleophile, reacts with carbonyl carbon of aldehyde to give anion **II**. Next, this anion takes a proton from protonated catalyst to deliver intermediate III. Finally, the desired product is formed after elimination of a water molecule.

## 4 Conclusion

A novel amine-containing magnetic periodic mesoporous organosilica with yolk-shell structure (YS-MPMO/pr-NH_2_) was successfully synthesized and characterized. The TGA, EDX and FT-IR analyses showed the successful immobilization/incorporation of propylamine and methylene groups into/onto material framework. The SEM image confirmed that the YS-MPMO/pr-NH_2_ has a spherical morphology. Also, the superparamagnetic behavior of the YS-MPMO/pr-NH_2_ nanocomposite was proved by VSM analysis. The nitrogen-sorption analysis showed the presence of a shell with high surface area for the designed nanocamposite. The PXRD analysis demonstrated high stability of Fe_3_O_4_ NPs during the catalyst preparation. Examination of the catalytic activity of YS-MPMO/pr-NH_2_ in the Knoevenagel reaction showed that this catalyst has an excellent performance in this process. The leaching test confirmed the heterogeneous nature of active catalytic sites under applied conditions. The catalyst was also recovered and reused several times with maintaining its efficiency.

## Data Availability

The original contributions presented in the study are included in the article/supplementary material, further inquiries can be directed to the corresponding author.
